# COPI is essential for Golgi cisternal maturation and dynamics

**DOI:** 10.1242/jcs.193367

**Published:** 2016-09-01

**Authors:** Midori Ishii, Yasuyuki Suda, Kazuo Kurokawa, Akihiko Nakano

**Affiliations:** 1 Live Cell Super-Resolution Imaging Research Team, RIKEN Center for Advanced Photonics, 2-1 Hirosawa, Wako, Saitama 351-0198, Japan; 2 Department of Biological Sciences, Graduate School of Science, The University of Tokyo, 7-3-1 Hongo, Bunkyo-ku, Tokyo 113-0033, Japan; 3 Laboratory of Molecular Cell Biology, Faculty of Medicine, University of Tsukuba, Tsukuba, Ibaraki 305-8575, Japan

**Keywords:** Golgi, COPI, Cisternal maturation

## Abstract

Proteins synthesized in the endoplasmic reticulum (ER) are transported to the Golgi and then sorted to their destinations. For their passage through the Golgi, one widely accepted mechanism is cisternal maturation. Cisternal maturation is fulfilled by the retrograde transport of Golgi-resident proteins from later to earlier cisternae, and candidate carriers for this retrograde transport are coat protein complex I (COPI)-coated vesicles. We examined the COPI function in cisternal maturation directly by 4D observation of the transmembrane Golgi-resident proteins in living yeast cells. COPI temperature-sensitive mutants and induced degradation of COPI proteins were used to knockdown COPI function. For both methods, inactivation of COPI subunits Ret1 and Sec21 markedly impaired the transition from cis to medial and to trans cisternae. Furthermore, the movement of cisternae within the cytoplasm was severely restricted when COPI subunits were depleted. Our results demonstrate the essential roles of COPI proteins in retrograde trafficking of the Golgi-resident proteins and dynamics of the Golgi cisternae.

## Abstract (precis)

**Highlighted Article:** Knockdown of COPI function restricts retrograde recycling of Golgi-resident proteins and markedly impairs the transition from cis to medial and to trans cisternae, as demonstrated in living yeast cells.

## INTRODUCTION

The Golgi is a central station of the membrane trafficking system in eukaryotic cells. It consists of flattened membrane-enclosed compartments, called cisternae, which are orderly differentiated in their functions and structures from cis to trans cisternae. The Golgi receives newly synthesized proteins from the endoplasmic reticulum (ER) at cis cisternae, and processes and glycosylates them as they proceed through the medial regions, and finally sorts them from trans cisternae and the trans-Golgi network (TGN) to their final destinations. These functions of the Golgi are evolutionarily conserved. However, the organization of the Golgi varies across cell types and species; higher animal and plant cells have stacked Golgi cisternae, whereas the budding yeast *Saccharomyces cerevisiae* has unstacked cisternae dispersed in the cytoplasm (Mowbrey and Dacks, 2009).

The mechanism of protein trafficking within the Golgi is a fundamental and intriguing question of cell biology. To explain anterograde transport of secretory cargo, the ‘cisternal maturation’ model is now widely accepted to explain the core mechanism for Golgi traffic (Glick and Luini, 2011; Glick and Nakano, 2009; Nakano and Luini, 2010). This model is based on the concept that Golgi cisternae progressively change their nature and work as anterograde carriers for secretory protein transport. The support for this model in mammalian cells was given by the observation that large aggregates of procollagen progressively moved through the Golgi stacks without leaving the lumen (Bonfanti et al., 1998). Maturation of the Golgi cisternae has also been directly observed in *S. cerevisiae* (Losev et al., 2006; Matsuura-Tokita et al., 2006). Individual early and late Golgi cisternae in *S. cerevisiae* were labeled with different fluorescent-protein-tagged Golgi-resident proteins and the colors of cisternae showed a unidirectional change from early to late under a confocal fluorescence microscope. In this view, Golgi-resident proteins should be transported from late to early cisternae by retrograde transport machineries. Furthermore, Rizzo et al. (2013) reported that artificial polymerization of Golgi-resident proteins to prevent recycling led to their progression through the Golgi stack, which also supports cisternal maturation (Rizzo et al., 2013). However, many questions remain as to its molecular mechanisms.

Coat protein complex I (COPI)-coated vesicles are the candidate retrograde transport carriers of Golgi-resident proteins. COPI coats are composed of seven subunits, known as α, β, β′, γ, δ, ε and ζ COP, which are classified into two groups: α, β′ and ε subunits forming the B trimeric adaptor complex, and β, γ, δ and ζ subunits forming the F tetrameric outer coat complex (Gabriely et al., 2007; Lee and Goldberg, 2010). A function of COPI-coated vesicles in anterograde cargo transport is still a matter of debate, however COPI has pivotal roles in Golgi–ER retrograde transport and probably also in the retrograde traffic between Golgi cisternae (Cosson et al., 2002; Emr et al., 2009; Martínez-Menárguez et al., 2001; Orci et al., 2000; Rabouille and Klumperman, 2005; Sato et al., 2001). Recruitment of COPI coat proteins to the Golgi membrane requires Arf GTPase (Serafini et al., 1991). A recent report has shown that disruption of Arf1 causes early Golgi cisternae to mature more slowly and less frequently, but does not alter the maturation of late Golgi cisternae in *S. cerevisiae* (Bhave et al., 2014).

We reported before that the α-COP temperature-sensitive mutant *ret1-1*, which is defective in COPI vesicle formation at the restrictive temperature, exhibited retarded but not completely blocked maturation from early to late cisternae at the restrictive temperature by 2D time-lapse observations (Matsuura-Tokita et al., 2006). From this result, we suggested that COPI is important for Golgi cisternal maturation, but other mechanisms might also operate. For technical reasons 3D observation was not performed, and we could not rule out the ambiguity of analysis from 2D data. To further clarify the role of COPI proteins in the cisternal maturation, we decided to revisit these results by using transmembrane Golgi-resident proteins as cis-, medial- and trans-cisternal markers and by a more-elaborate 3D observation by high-speed and high-resolution confocal microscopy method that we developed (super-resolution confocal live imaging microscopy, SCLIM) (Kurokawa et al., 2013). To inactivate COPI functions, not only temperature-sensitive defects of COPI proteins, but also a new method to induce their degradation by an auxin degron system (Nishimura et al., 2009) was employed. We found that disruption of COPI functions inhibited cisternal maturation and dynamic movement of cisternae in the cytoplasm. These results indicate that the retrograde transport of the Golgi-resident proteins is mediated through a COPI-dependent mechanism that plays a pivotal role in the cisternal maturation, and that COPI proteins also play some role in the Golgi dynamics.

## RESULTS

### Spatial distribution of transmembrane Golgi-resident proteins

**Fig. 1.**
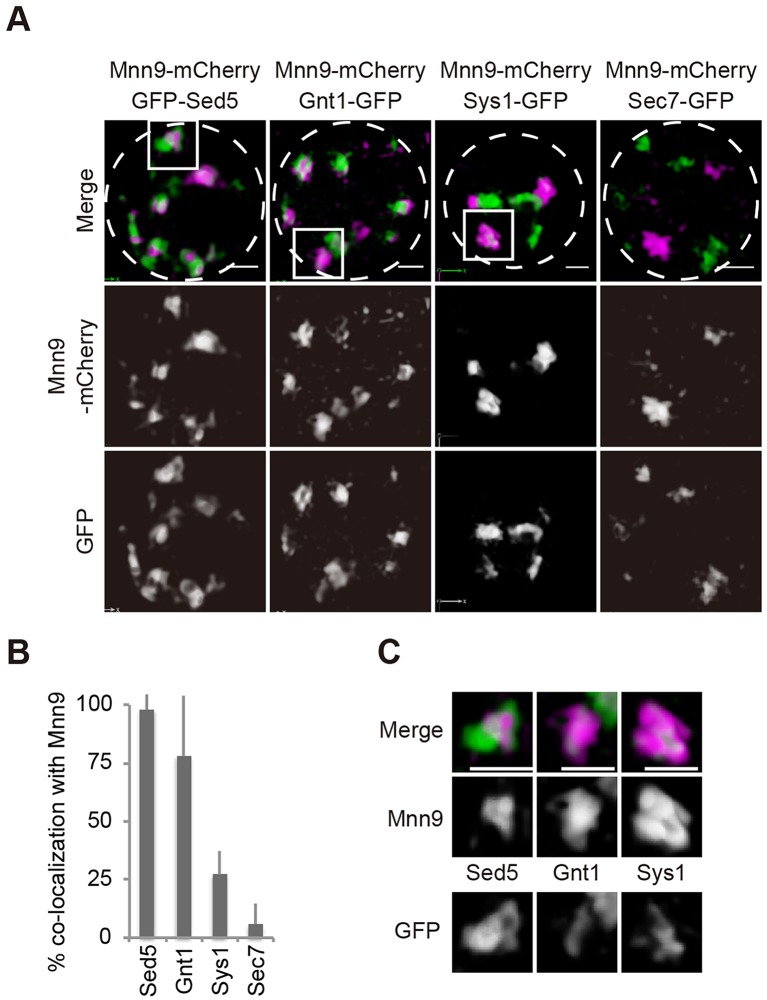
**Localization of Golgi-resident proteins.** (A) Localization of Golgi marker proteins with the cis-Golgi-resident marker Mnn9–mCherry. Wild-type cells expressing GFP-tagged Sed5 (cis), Gnt1 (medial), Sys1 (trans) and Sec7 (trans) with Mnn9–mCherry were grown to a mid-logarithmic phase in synthetic medium at 30°C and observed by 3D confocal fluorescence microscopy. Dashed lines indicate the edge of the cells. (B) Bar graph showing the percentage of Mnn9–mCherry-positive cisternae containing GFP-tagged Golgi-resident proteins. Error bars indicate s.d. from 10 independent cells. (C) Images of indicated areas in A. Scale bars: 1 µm.

A variety of fluorescent-tagged proteins have been used for live imaging of yeast Golgi cisternae (Matsuura-Tokita et al., 2006). Here, we employ as new markers, Golgi-resident proteins harboring transmembrane domains, which are Mnn9, a subunit of Golgi mamnosyltransferase complex, Gnt1, an *N*-acetylglucosaminyltransferase, and Sys1, a receptor for Arl3, for cis, medial, and trans cisternae, respectively (Behnia et al., 2004; Jungmann and Munro, 1998; Yoko-o et al., 2003). Mnn9 and Gnt1 are type II transmembrane glycosyltransferases and have a Vps74 recognition motif at their N-termini. Vps74 packs them into COPI-coated vesicles for recycling to early cisternae and controls their steady-state distributions (Tu et al., 2008). Sys1 has been shown to act as a transmembrane receptor for Arl3, but its retention is independent of Vps74 (Schmitz et al., 2008). These resident proteins and commonly used Golgi cisternae markers, Sed5, a t-soluble *N*-ethylmaleimide-sensitive factor attachment protein receptor (SNARE) molecule mostly reside in the cis cisternae (Hardwick and Pelham, 1992), and Sec7, the guanine-nucleotide exchange factor for Arf GTPase, which is peripherally associated with trans cisternae and TGN (Achstetter et al., 1988), were tagged with green or red fluorescent proteins. The degrees of colocalization of these Golgi marker proteins were examined by dual-color 3D observation (SCLIM). As shown in Fig. 1A, the two cis markers GFP–Sed5 and Mnn9–mCherry showed a very high probability of colocalization. The percentage colocalization with Mnn9–mCherry for Gnt1–GFP, Sys1–GFP and Sec7–GFP was ∼77%, ∼26% and ∼5%, respectively, consistent with their medial- and trans-cisternal localization (Fig. 1A,B). These results reflect the preferential distribution of Golgi-resident proteins to particular Golgi cisternae. Note that segregation of two markers is observed within a colocalizing cisterna by SCLIM (Fig. 1C).

### Visualization of cisternal maturation using transmembrane Golgi-resident proteins

**Fig. 2.**
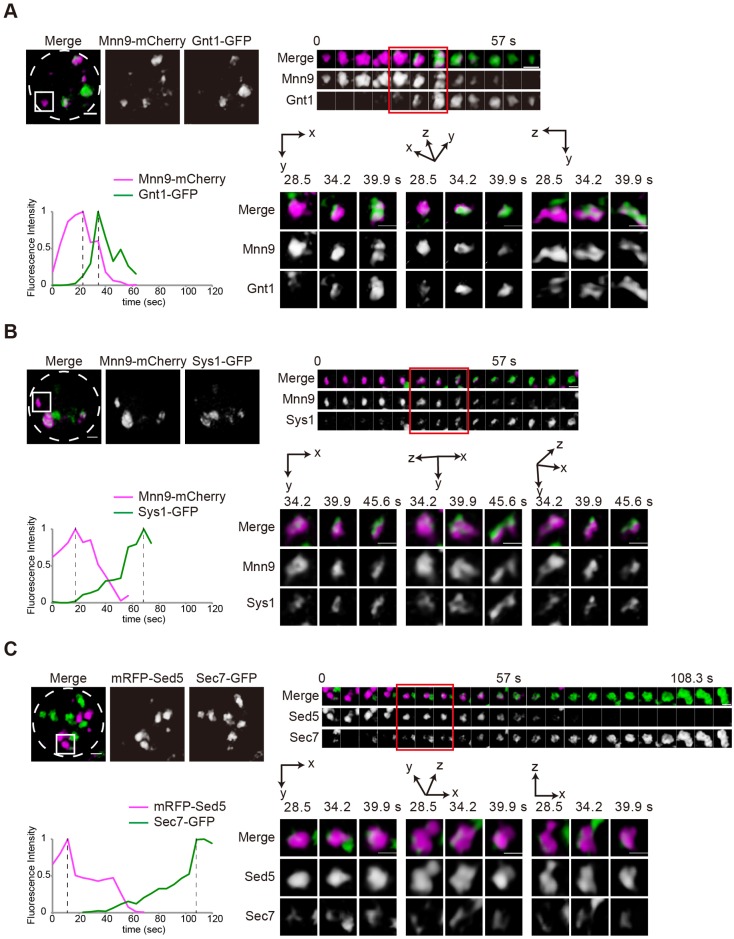
**4D observation of cisternal maturation.** Wild-type cells expressing (A) Mnn9–mCherry (cis, magenta) and Gnt1–GFP (medial, green), (B) Mnn9–mCherry (cis, magenta) and Sys1–GFP (trans, green) and (C) mRFP–Sed5 (cis, magenta) and Sec7–GFP (trans, green) were grown to a mid-logarithmic phase in synthetic medium at 30°C and observed by SCLIM. Upper left panels show representative 3D images. Dashed lines indicate the edge of the cells. Upper right montages show 3D time-lapse images (4D) of the indicated squares. Lower left panels show the relative fluorescence intensities of green and red channels in the indicated cisterna. Lower right panels show magnified images of cells at selected time points. Scale bars: 1 µm. Representative images from at least 26 independent cisternae are shown.

We next examined cisternal maturation of the Golgi in detail by using these Golgi-resident markers. We conducted simultaneous dual-color 4D observations at time resolution of 8 frames/s by SCLIM (Kurokawa et al., 2013; Matsuura-Tokita et al., 2006). Similar to the case in the cell expressing mRFP–Sed5 and Sec7–GFP (Fig. 2C and Movie 3), early cisternae labeled with Mnn9–mCherry changed their color to those of medial cisternae labeled with Gnt1–GFP or trans cisternae and TGN labeled with Sys1–GFP, giving evidence for cisternal maturation through recycling of Golgi-resident transmembrane proteins (Fig. 2A,B; Movies 1 and 2). We determined the transition period between the intensity peaks for each pair of fluorescent markers on a single cisterna (peak-to-peak time) (Daboussi et al., 2012). The average peak-to-peak time from Mnn9–mCherry (cis) to Gnt1–GFP (medial) (38.6±11.6 s) was shorter than that from Mnn9–mCherry (cis) to Sys1–GFP (trans) (45.6±19.6 s) (mean±s.d., *n*>11; Fig. 2A,B). The transition period from mRFP–Sed5 (cis) and Sec7–GFP (trans) (87.8±34.0 s) was longer than that from Mnn9–mCherry (cis) to Sys1–GFP (trans) (Fig. 2C). Even though experimental variability was high, these results were consistent with the spatially ordered distribution of Golgi-resident proteins within the Golgi. High-resolution images during the transition of two fluorescent signals revealed that later markers of the Golgi cisternae, Gnt1–GFP, Sys1–GFP and Sec7–GFP, began to accumulate as small punctate structures on the early cisternae and increased their volume to cover entire regions on the membrane. By contrast, the earlier markers, Mnn9–mCherry and mRFP–Sed5 signals showed a gradual reduction (Fig. 2A–C). In addition, we noticed that earlier and later Golgi markers did not completely overlap but rather showed segregation within the maturing cisternae, corroborating that functional domains exist within a Golgi cisterna (see also Fig. 1C). We could not observe transition from Mnn9–mCherry-labeled cis cisternae to Sec7–GFP-labeled trans cisternae, indicating that Sec7–GFP begins to accumulate later than the disappearance of Mnn9–mCherry from cisternae. This is consistent with the above finding that Mnn9 rarely colocalized with Sec7 (Fig. 1A,B; Fig. S1). The two trans markers Sys1 and Sec7 probably accumulate on cisternae at different maturation phases; Sec7 comes later than Sys1.

### 4D observation of cisternal maturation in temperature-sensitive COPI mutant cells

In our previous report, we showed that cisternal maturation was slowed down but was still observed in α-COP temperature-sensitive mutant *ret1-1* cells at the restrictive temperature (Matsuura-Tokita et al., 2006). Because this previous observation was made only in 2D time lapse, we decided to reinvestigate this issue using our much improved 4D imaging system, SCLIM, which has high sensitivity and high resolution.

**Fig. 3.**
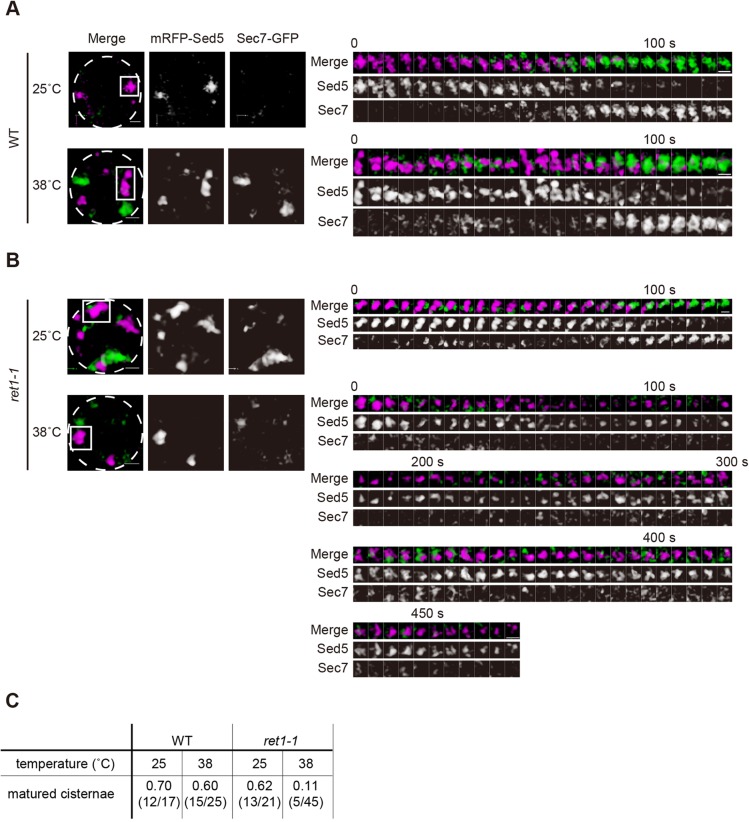
**4D observation reveals the defect of cisternal maturation in the α-COP mutant *ret1-1* at the restrictive temperature.** Wild-type (WT) and *ret1-1* cells expressing mRFP–Sed5 (cis, magenta) and Sec7–GFP (trans, green) were grown to a mid-logarithmic phase in synthetic medium at 25°C. Wild-type cells cultured at 25°C and 38°C for 10 min (A) and *ret1-1* cells cultured at 25°C (permissive) and 38°C (restrictive) temperature for 10 min (B) were observed by SCLIM. Representative 3D images are shown. Dashed lines indicate the edge of the cells. Right montages show 3D time-lapse (4D) images of the indicated squares. Scale bars: 1 µm. (C) The numbers of cisternae that had matured are shown. At least independent 17 cisternae were counted.

Both in wild-type and *ret1-1* cells at 25°C, transition of the fluorescent signals from mRFP–Sed5 to Sec7–GFP was observed within 120 s, indicating that efficient cisternal maturation occurred in the cells (Fig. 3A,B). By contrast, in *ret1-1* cells at the restrictive temperature, cis cisternae labeled with mRFP–Sed5 did not lose red fluorescence but kept it for more than 400 s, and never acquired Sec7–GFP signals (Fig. 3B; Movie 4). The rate of successful Golgi maturation was greatly decreased (11%, 5 out of 55 cisternae) in *ret1-1* cells at the restrictive temperature compared to the permissive temperature (62%, 13 out of 21 cisternae). No significant difference was observed for the wild-type upon temperature shift (Fig. 3C). These observations indicate that COPI function is indispensable for cisternal maturation.

### 4D observation of cisternal maturation in cells depleted for COPI proteins

**Fig. 4.**
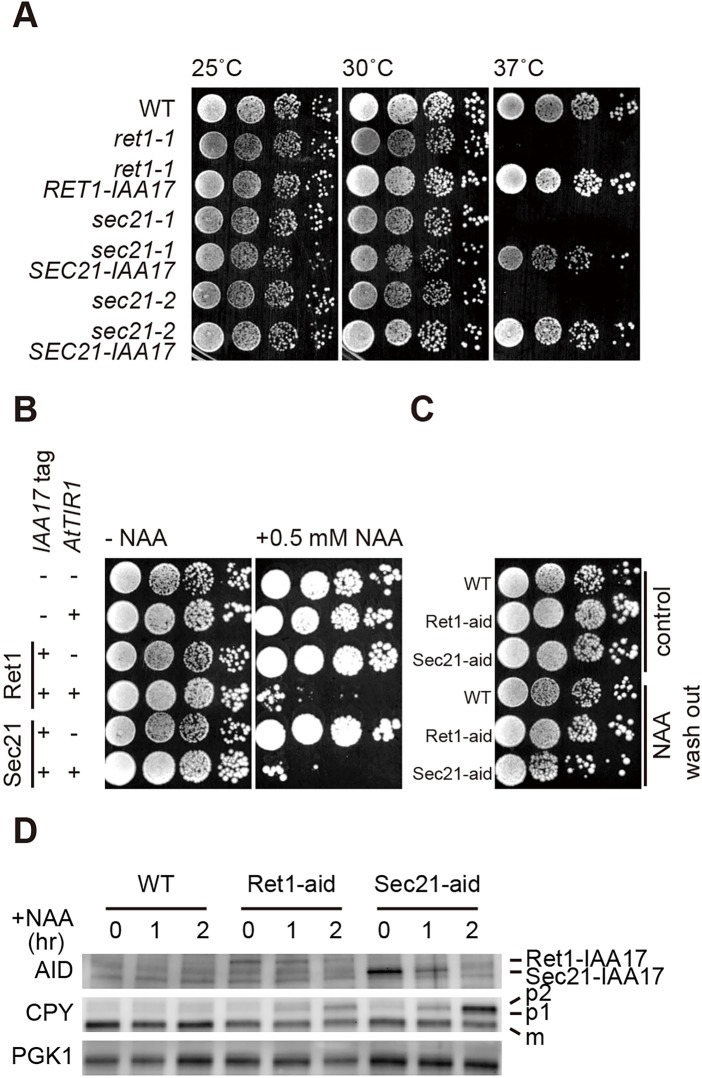
**Degradation of the COPI proteins Ret1 and Sec21 by the AID system.** (A) Expression of IAA17-tagged Ret1 and Sec21 from low-copy plasmids rescued the temperature-sensitive phenotypes of *ret1-1* and *sec21-1* or *sec21-2* cells, respectively. (B) Growth of wild-type (WT), AtTIR1, Ret1-IAA17, Ret1-aid, Sec21-IAA17 and Sec21-aid cells on a YPD plate with or without 0.5 mM NAA at 30°C. (C) Growth of NAA-treated cells after NAA washout. Wild-type, Ret1-aid and Sec21-aid cells were cultured in YPD medium with or without 1 mM NAA for 2 h at 30°C. After washing out medium with water three times, cells were grown on a YPD plate at 30°C. (D) Ret1-aid and Sec21-aid cells were cultured in YPD medium with or without 1 mM NAA at 30°C. Cells were collected at 0, 1 and 2 h after NAA addition. Ret1-IAA17, Sec21-IAA17 and CPY proteins were detected by immunoblotting. The positions of the ER form (p1), Golgi form (p2) and mature (m) CPY are indicated on the right. The PGK lane shows the loading control. All experiments were performed three times and a representative example shown.

To further elucidate the role of COPI proteins in cisternal maturation, we developed cells in which COPI proteins were degraded through an auxin-inducible degron (AID) system. The AID system allows specific protein degradation in the presence of plant hormone auxin in non-plant cells (Nishimura et al., 2009). *Arabidopsis thaliana* E3 ubiquitin ligase TIR1 was constitutively expressed in yeast cells and the *A. thaliana* IAA17 sequence was added as a tag at the C-terminus of Ret1 (α-COP) or Sec21 (γ-COP). These cells are designated Ret1-aid and Sec21-aid cells, respectively, or COPI-aid cells, collectively. Expression of IAA17-tagged Ret1 and Sec21 could rescue the growth defects of corresponding temperature-sensitive mutants at the restrictive temperature (Fig. 4A). COPI-aid cells exhibited strong growth defects in the presence of 0.5 mM 1-naphthaleneacetic acid (NAA, a synthetic auxin), whereas they grew normally as wild-type cells in the absence of NAA (Fig. 4B). More than 80% of IAA17-tagged COPI proteins were degraded upon incubation with 1 mM NAA for 2 h (Fig. 4D). Auxin-dependent degradation of COPI proteins was reversible, because the growth rates of COPI-aid cells after NAA washout were comparable to that of wild-type cells (Fig. 4C). ER and Golgi precursor forms of a vacuolar protein, carboxypeptidase Y (CPY), accumulated in COPI-aid cells after NAA treatment for 2 h (Fig. 4D).

**Fig. 5.**
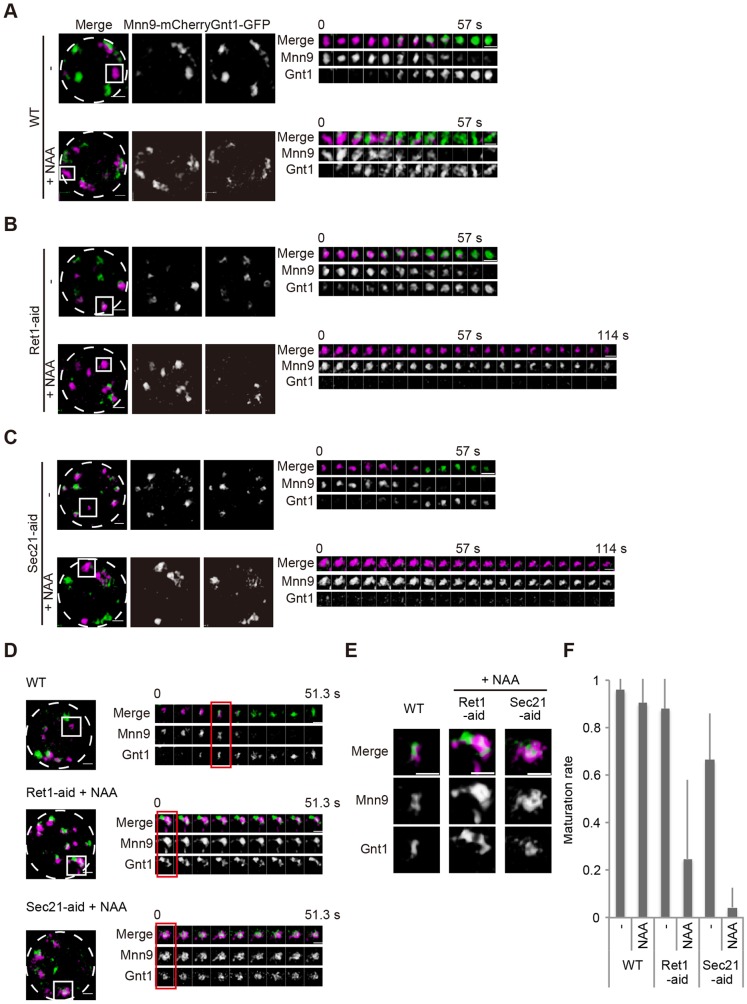
**Depletion of COPI protein inhibits cis to medial cisternal maturation.** (A) Wild-type (WT), (B) Ret1*-*aid and (C) Sec21-aid cells expressing Mnn9–mCherry (cis, magenta) and Gnt1–GFP (medial, green) were grown to a mid-logarithmic phase in synthetic medium with or without 1 mM NAA at 30°C and observed by SCLIM. Representative 3D images of cells with (lower panels) or without (upper) NAA are shown. Dashed lines indicate the edge of cells. Right montages show 3D time-lapse images of the indicated areas. (D) Wild-type, Ret1*-*aid and Sec21*-*aid cells with NAA expressing Mnn9–mCherry (cis, magenta) and Gnt1–GFP (medial, green) were observed. Left panels show representative 3D images of cells. Dashed lines indicate the edge of cells. Right montages show 3D time-lapse images of the indicated areas. (E) Magnified images of selected time points in D are shown. Scale bars: 1 µm. (F) Bar graph shows the rate of maturation of cisternae from cis to medial. Error bars represent s.d. from at least five independent cells.

**Fig. 6.**
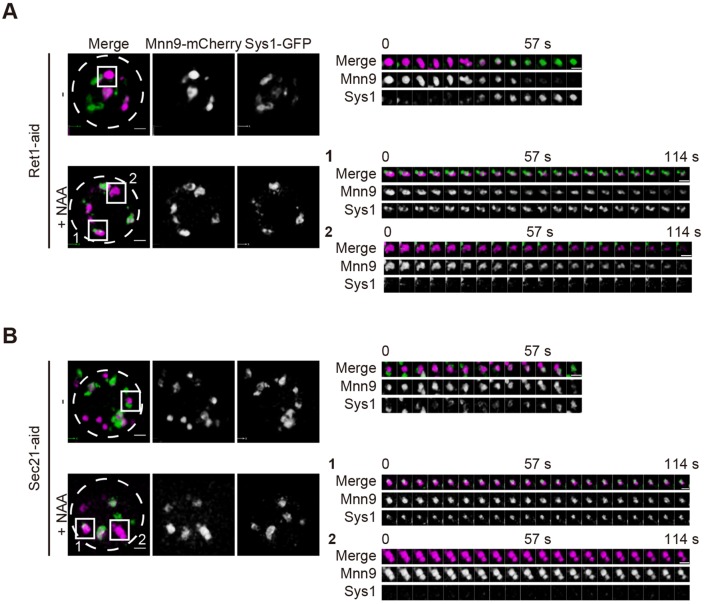
**Depletion of COPI protein inhibits cis to trans cisternal maturation.** Ret1*-*aid (A) and Sec21-aid (B) cells expressing Mnn9–mCherry (cis, magenta) and Gnt1–GFP (medial, green) were grown to a mid-logarithmic phase in synthetic medium with or without 1 mM NAA at 30°C and observed by SCLIM. Representative 3D images of cells with (lower panels) or without (upper) NAA are shown. Dashed lines indicate the edge of cells. Right panels show 3D time-lapse images of the indicated areas. Scale bars: 1 µm. Representative images from at least five independent cells are shown.

Using COPI-aid cells expressing Mnn9–mCherry and Gnt1–GFP, we next examined the effect of COPI protein removal on cisternal maturation. In COPI-aid cells treated with 1 mM NAA for 2 h, cisternae with the signal of Mnn9-mCherry were sustained and never acquired the Gnt1-GFP signal (Fig. 5A–C; Movies 5 and 6). The rates of cisternal maturation in COPI-aid cells were significantly reduced by NAA treatment (Fig. 5F). These observations again indicate that COPI function is required for the retrograde flux of Golgi-resident proteins and is essential for cisternal maturation of the Golgi. Intriguingly, functional domains labeled with Mnn9–mCherry and Gnt1–GFP were kept segregated within the cisterna in NAA-treated COPI-aid cells (Fig. 5D,E), suggesting that COPI function is also necessary for domain dynamics. We next examined the effect of COPI depletion on the behaviors of the trans-Golgi and TGN marker Sys1–GFP. Transition from Mnn9–mCherry to Sys1–GFP was also disturbed in the presence of NAA in COPI-aid cells (Fig. 6). Cis cisternae labeled with Mnn9–mCherry never lost their color (Fig. 6A,B, boxed area 2; Movies 7 and 8). Cisternae harboring both Mnn9–mCherry and Sys1–GFP sustained both fluorescent signals for a prolonged time (Fig. 6A,B, boxed area 1; Movies 7 and 8). These results indicate that the function of COPI is required over the whole maturation process of cis-to-trans cisternae within the Golgi.

### Reduced movements of the Golgi in COPI inactivated cells

**Fig. 7.**
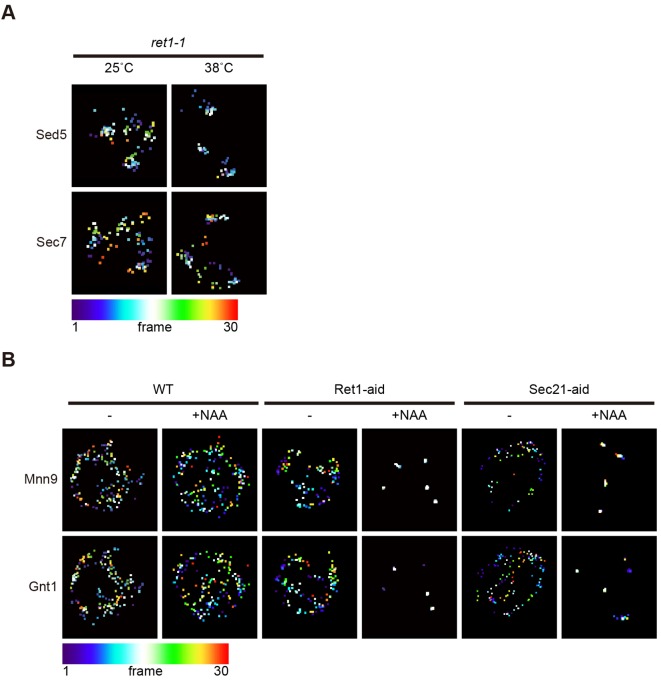
**Inhibition of COPI functions reduced cisternal movement.** The *z* and time projections of the center of mass of each cisterna are shown. The colors of points indicate the time point shown at the bottom left. (A) Images of *ret1-1* cells expressing mRFP–Sed5 (cis) and Sec7–GFP (trans) at 25°C and 38°C were processed. One *z*-stack was collected every 5 s. (B) Images of wild-type (WT), Ret1-aid and Sec21-aid cells expressing Mnn9–mCherry (cis) and Gnt1–GFP (medial) with or without 1 mM NAA for 2 h were processed. *z*-stack images were collected for every 5.7 s. Representative images from at least five independent cells are shown.

In the course of our observation of cisternal maturation in *ret1-1* cells and COPI-aid cells, we realized that the dynamics of Golgi cisternae was affected upon COPI disruption (Fig. 7). *S. cerevisiae* has single layers of Golgi cisternae scattering and moving around in the cytoplasm. We have recently discovered that the cis cisternae of Golgi show repeated approach toward ER exit sites (ERES) (hug-and-kiss action) (Kurokawa et al., 2014) to capture cargo from the ER. In *ret1-1* cells at restrictive temperature, the movement of Sed5-labeled cis cisternae was drastically restricted, whereas that of Sec7-labeled trans cisternae was less affected (Fig. 7A; Movie 4). In NAA-treated COPI-aid cells, 3D tracking of cis and medial cisternae demonstrated remarkable inhibition of the cisternal movement (Fig. 7B; Movies 5 and 6). The structures of the ER did not appear to be affected under these conditions (Fig. S2). Disruption of actin or microtubule cytoskeletons sensitive to latrunculin A and nocodazole, respectively, did not affect either cisternal maturation or movement (Fig. S3). These results suggest that COPI contributes to the dynamic behavior of Golgi cisternae in *S. cerevisiae*.

## DISCUSSION

### COPI-dependent recycling of Golgi-resident proteins drives cisternal maturation from cis to trans

Cisternal maturation explains a core mechanism for intra-Golgi traffic (Losev et al., 2006; Luini, 2011; Matsuura-Tokita et al., 2006). In a typical model, Golgi cisternae serve as anterograde carriers for secretory cargo, and the retrograde transport of Golgi-resident proteins executes the change in properties of cisternae called maturation. Direct evidence for cisternal maturation was provided by live imaging of yeast *S. cerevisiae* using fluorescent-protein-tagged Golgi-resident proteins (Losev et al., 2006; Matsuura-Tokita et al., 2006). We were aware, however, of a caveat in selection of fluorescent marker proteins. For example, a frequently used trans-Golgi marker, Sec7, is a peripheral membrane protein and its recycling between the cytosol and the membrane cannot be ruled out. SNARE proteins are also convenient markers but as they constitute important transport machinery their behaviors may not represent ‘resident’ Golgi proteins. In the present study, we have tried to exploit as new markers fluorescence-tagged Golgi-resident transmembrane proteins, such as enzymes, and confirmed that they are also good tools to analyze cisternal maturation. By using SCLIM imaging technology that we developed, we now provide 4D observation of these fluorescence Golgi cisternal markers at high spatiotemporal resolutions (Kurokawa et al., 2013). We addressed the roles of COPI in intra-Golgi transport processes, and the results indicate that the COPI functions are essential for fulfilling cisternal maturation. Both a temperature-sensitive defect (*ret1-1* mutation) and auxin-induced degradation of Ret1 or Sec21 led to severe blockade of cisternal maturation in cis-to-medial and medial-to-trans steps.

Whether COPI executes its functions in the form of COPI vesicles is still an open question. COPI is also involved in tubule formation in mammalian cells (Yang et al., 2011). We sometimes see tubular connections between yeast Golgi cisternae (Matsuura-Tokita et al., 2006; Nakano and Luini, 2010). Our observation in the present study that small membrane puncta from later cisternae emerge on a maturing cisterna (discussed below) strongly suggests the presence of vesicles there, but at the moment we cannot conclude whether they are single vesicles or clusters of vesicles. Roles for COPI-coated tubules are also possible. We are now in the process of developing a second-generation SCLIM system, which has even better spatiotemporal resolutions, and we hope to clarify these questions in the near future.

Another important question is how COPI controls selective recycling of distinct Golgi-resident proteins to their own cisternae. One of the candidates for regulating the selectivity to specific cisternae would be golgin family proteins, because they are thought to specifically capture vesicles from different organelles, endosomes, the ER, and the Golgi. For example, Golgin-84 (also known as GOLGA5), GMAP210 (also known as TRIP11) and TMF1 have been shown to capture the Golgi-resident proteins in mammalian cells (Wong and Munro, 2014). In yeast, Rud3 and Sgm1, orthologs of GMAP210 and TMF1, localize to cis and trans cisternae, respectively (Munro, 2011). Whether selective degradation of these golgin family proteins by the AID system affects intra-Golgi trafficking of resident proteins would be a next question to be examined by our high-resolution 4D observation.

### Domains are formed and maintained on Golgi cisternae during cisternal maturation

During cisternal maturation, segregation of early and late Golgi-resident proteins was observed in individual cisternae in our previous report (Matsuura-Tokita et al., 2006). Here, our 4D imaging with higher spatiotemporal resolutions reveals details of the compositional change in maturing individual cisternae (see Fig. 2). At the beginning of the transition of cis cisternae, marked by Mnn9–mCherry, Gnt1–GFP coming from medial cisternae began to localize to small regions on the cis cisternae. These small puncta gradually expanded their area without extensive mixing with the cis component and eventually covered the whole cisternae. Similar events were also observed for the cases of Sys1–GFP and Sec7–GFP coming into the cis-cisternae. Furthermore, two cis-Golgi marker proteins, Sed5 and Mnn9, showed segregated distribution on a single cisterna (Fig. 1C). Such segregation of membrane proteins might be an intrinsic property of the Golgi membrane and would be a key to understand their sorting and dynamic equilibrium. In COPI-depleted cells, domain segregation was still observed but in a fixed fashion (see Figs 5 and 6), suggesting the involvement of COPI in dynamic mixing of domains. The Rab family of GTPases regulate a variety of steps of membrane trafficking. They show phase-specific localization on the Golgi membrane, which is coordinately controlled by Rab GEF and Rab GAP cascades (Rivera-Molina and Novick, 2009; Suda et al., 2013). We previously showed that the cells disrupted for the Rab GAP cascade failed in proper Rab GTPase transition and thus in proper membrane segregation but somehow continued cisternal maturation (Suda et al., 2013). The significance of membrane domain segregation will need further investigation.

### COPI also functions in dynamics of Golgi cisternae

Recently, Glick and colleagues showed that inactivation of COPI function by anchoring COPI subunits on mitochondria led to the formation of hybrid Golgi structures, in which both early (Vrg4–GFP) and late (Sec7–DsRed or Kex2–mCherry) Golgi-resident proteins were present (Papanikou et al., 2015). These structures fluctuated rapidly and moved around in the cytoplasm. They maintained the early Golgi marker, but showed repeated loss and gain of late Golgi and TGN makers. Anchored COPI proteins have potential to still interact to the Golgi. In our present study by contrast, the AID system directly removes COPI proteins from cells and COPI depletion brought about marked inhibition of maturation of cis-, medial- and trans-Golgi and TGN markers (Figs 5 and 6) as well as remarkable repression of the dynamic movement of Golgi cisternae (Fig. 7). Cisternae labeled with both cis-Golgi and medial-Golgi or trans-Golgi and TGN markers might be deemed similar to the hybrid Golgi structures of Papanikou et al. (2015). However, they sustained these fluorescent signals and did not show repeated loss and gain behavior in our SCLIM observation. Colocalization of both early and late Golgi-resident proteins in a single cisterna was observed as only transient events during cisternal maturation in wild-type cells. These results suggest that in COPI-depleted cells the amount of COPI proteins is insufficient to recycle Golgi-resident proteins, resulting in intermediate cisternae becoming fixed in the maturation process.

Why the dynamics of cisternae is amazingly disturbed by COPI depletion remains a big question to be addressed. Perhaps dynamic release of COPI vesicles or projection of COPI tubules can affect the balance of individual cisternae. To test such a possibility mathematical modeling might be helpful. Recently, we have shown that cis-Golgi cisternae show repeated approach toward the COPII-coated area localized at ERES and capture cargo proteins there (hug-and-kiss action; Kurokawa et al., 2014). The dynamic behavior of Golgi cisternae must be a pivotal feature in *S. cerevisiae* that plays many roles in controlling anterograde and retrograde transport in secretory pathway. Analysis of COPI functions would be again a key to tackle the questions of Golgi dynamics.

## MATERIALS AND METHODS

### Yeast strains and plasmids

Yeast strains, plasmids and primers used in this study are listed in Tables S1, S2 and S3, respectively. *ADE2+* cells were made by integration with pRS402 (Brachmann et al., 1998) digested by *Stu*I into the *ade2* site. mRFP–Sed5 and Sec7–GFP were co-expressed under the control of the *TDH3* promoter for mRFP–Sed5 and the *ADH1* promoter for Sec7–GFP on the low-copy plasmid pRS316 (Matsuura-Tokita et al., 2006). Gnt1–GFP and Sys1–GFP were expressed under the control of *ADH1* promoter and *CMK1* terminator on the low-copy plasmids pRS316 or pRS314 (Sikorski and Hieter, 1989). These plasmids were constructed in several steps. First, the DNA fragment containing the *ADH1* promoter or *CMK1* terminator was obtained from yeast genomic DNA by PCR with an appropriate sets of primers, SacI-ADH1p-F and ADH1p-NotI-R, or XhoI-CMK1t-F and CMK1t-SacI-KpnI-R, was subcloned into the *Sac*I-*Not*I or *Xho*I-*Kpn*I sites of pRS316, respectively. Next, the *GFP* DNA fragment, obtained from pSKY5 (Sato et al., 2001) by PCR with primers SalI-GFP-F and GFP-XhoI-R, was subcloned into the *Sal*I-*Xho*I sites. Then, the *GNT1* or *SYS1* DNA fragments obtained from yeast genomic DNA by PCR with primers NotI-GNT1-F and GNT1-SalI-R or XbaI-SYS1-F and SYS1-HindIII-R were subcloned into the *Not*I-*Sal*I sites or *Xba*I-*Hin*dIII sites of pRS316 harboring the *ADH1* promoter, *GFP* and the *CMK1* terminator to develop pRS316-ADH1p-GNT1-GFP-CMK1t or pRS316-ADH1p-SYS1-GFP-CMK1t, respectively. pRS316-ADH1p-GNT1-GFP-CMK1t was digested by *Sac*I and subcloned into the *Sac*I site of pRS314 to produce pRS314-ADH1p-GNT1-GFP-CMK1t. pRS316-ADH1p-SYS1-GFP-CMK1t was digested by *Pvu*II and subcloned between the *Pvu*II sites of pRS314 to produce pRS314-ADH1p-SYS1-GFP-CMK1t. pRS304-SEC71TMD-GFP was constructed by subcloning the DNA fragment between *Pvu*II sites of pRS306-SEC71TMD-GFP (Sato et al., 2003) into pRS304 (Sikorski and Hieter, 1989). Strains expressing Sec71TMD-GFP were constructed by the integration with DNA fragment of pRS304-SEC71TMD-GFP digested by *Bsu*36I into the *trp1* site of yeast genome. Strains expressing fluorescent-protein-tagged Mnn9 were constructed by a PCR-based method described in a previous report (Kurokawa et al., 2014).

Strains expressing IAA17-tagged Ret1 and Sec21 at their C-termini were constructed by a PCR-based method using pMK43 plasmid as a template (Nishimura et al., 2009) and the primers listed in Table S3 (RET1-S2 and RET1-S3 for aid-tagged *RET1* and SEC21-S2 and SEC21-S3 for aid-tagged *SEC21*). *Arabidopsis thaliana* E3 ubiquitin ligase TIR1 was expressed from pMK76 integrated into the *ura3* site of each yeast genome by *Stu*I digestion (Nishimura et al., 2009).

### Microscopy

Fluorescence microscopy was performed by super-resolution confocal microscopy (SCLIM). The system setup had an Olympus model IX-71 inverted fluorescence microscope with a UPlanSApo 100×NA 1.4 oil objective lens (Olympus, Japan), a high-speed spinning-disk confocal scanner (Yokogawa Electric, Japan), a custom-made spectroscopic unit, image intensifiers (Hamamatsu Photonics, Japan) with a custom-made cooling system, and two EM-CCD cameras (Hamamatsu Photonics, Japan) for green and red channels (Kurokawa et al., 2013). For microscopic observation, all strains were grown in selective medium (0.67% yeast nitrogen base without amino acids and 2% glucose) with appropriate supplements. *ret1-1* mutant cells were cultured at the permissive temperature (25°C) and then incubated at the restrictive temperature (38°C) for 10 min on a thermo-controlled stage (Tokai Hit, Japan) whose temperature was kept at 38°C. Ret1-aid cells and Sec21-aid cells were grown with 1 mM NAA for 2 h. For 4D live imaging of wild-type cells, Ret1-aid cells and Sec21-aid cells, 32 optical slices spaced 0.1 µm apart were sequentially collected at 8 frames per second (fps), which takes 5.7 s in total including the time for data transfer. For 4D observation of *ret1-1* mutant cells, 32 optical slices 0.1 µm apart were collected at 15 fps every 5 s. *z*-stack images were converted to 3D voxel data and processed by iterative deconvolution with Volocity (Perkin Elmer) using a theoretical point-spread function for spinning-disc confocal microscopy. Maximum intensity projection images from these deconvolved 3D images were used for the calculation of the relative fluorescent values of the green and red signals of the Golgi areas with Fiji software (Schindelin et al., 2012). The center of mass of each cisterna was calculated in a mask of the cisterna from raw 4D images by Fiji software. The projection of the center of mass was conducted by Temporal-Color Code of Fiji plugin with a thermal lookup table.

### Immunoblotting

Cells expressing AID-tagged proteins were cultured in YPD (1% yeast extract, 2% peptone, and 2% glucose) medium at 30°C for overnight. Yeast culture was inoculated in fresh medium and grown until the optical density at 600 nm (OD_600_) was 0.5. 0.5 OD_600_ unit cells were collected for the loading sample. Cultures were incubated at 30°C for 1 or 2 h with or without 1 mM NAA, and were collected for loading samples at 1 or 2 h with or without NAA. 0.5 OD_600_ unit cells were suspended in 100 µl Laemmli's sample buffer and then disrupted by vortexing with glass beads. The cell suspensions were boiled at 100°C for 5 min and cleared by centrifugation at 20,000 ***g*** for 5 min to prepare total cell lysates. Proteins corresponding to 5 µl of total cell lysates were analyzed by SDS/PAGE, followed by western blotting with anti-AID antibody (1:2000, BioROIS), anti-CPY antibody (1:3000, rabbit, polyclonal) (Yahara et al., 2001) and anti-PGK antibody (1:10,000, Invitrogen, 22C5). Bands were visualized by horseradish-peroxidase-conjugated sheep anti-mouse IgG and donkey anti-rabbit IgG antibodies (GE Healthcare).
